# Phytochemical Screening, In Vitro and In Silico Studies of Volatile Compounds from *Petroselinum crispum* (Mill) Leaves Grown in Saudi Arabia

**DOI:** 10.3390/molecules27030934

**Published:** 2022-01-29

**Authors:** Ahmed I. Foudah, Mohammad H. Alqarni, Aftab Alam, Mohammad Ayman Salkini, Samir A. Ross, Hasan S. Yusufoglu

**Affiliations:** 1 Department of Pharmacognosy, College of Pharmacy, Prince Sattam Bin Abdulaziz University, Al Kharj 11942, Saudi Arabia; m.alqarni@psau.edu.sa (M.H.A.); a.alam@psau.edu.sa (A.A.); m.salkini@psau.edu.sa (M.A.S.); 2 National Center for Natural Products Research, School of Pharmacy, The University of Mississippi, University, MS 38677, USA; sross@olemiss.edu; 3 Department of Biomolecular Sciences, School of Pharmacy, The University of Mississippi, University, MS 38677, USA; 4 Department of Pharmacognosy & Pharmaceutical Chemistry, College of Dentistry & Pharmacy, Buraydah Private College, Buraydah 81418, Saudi Arabia; hasan.yusuf@bpc.edu.sa

**Keywords:** *Petroselinum crispum*, essential oil, antimicrobial agent, anti-inflammatory agent, in silico prediction, molecular docking

## Abstract

The herbal plant *Petroselinum crispum* (*P. crispum*) (Mill) is commonly available around the world. In this study, the leaves of the herbal plant *P. crispum* were collected from the central region of Al-Kharj, Saudi Arabia, to explore their in vitro pharmacological activity. Essential oil from the leaves of *P. crispum* was isolated using the hydrodistillation method. The composition of *P. crispum* essential oil (PCEO) was determined using Gas chromatography-mass spectrometry (GC-MS). A total of 67 components were identified, representing approximately 96.02% of the total volatile composition. Myristicin was identified as the principal constituent (41.45%). The in vitro biological activity was assessed to evaluate the antioxidant, antimicrobial, and anti-inflammatory potential of PCEO. PCEO showed the highest antimicrobial activity against *Candida albicans* and *Staphylococcus aureus* among all the evaluated microbial species. In vitro anti-inflammatory evaluation using albumin and trypsin assays showed the excellent anti-inflammatory potential of PCEO compared to the standard drugs. An in silico study of the primary PCEO compound was conducted using online tools such as PASS, Swiss ADME, and Molecular docking. In silico PASS prediction results supported our in vitro findings. Swiss ADME revealed the drug likeness and safety properties of the major metabolites present in PCEO. Molecular docking results were obtained by studying the interaction of Myristicin with an antifungal (PDB: 1IYL and 3LD6), antibacterial (PDB: 1AJ6 and 1JIJ), antioxidant (PDB: 3NM8 and 1HD2), and anti-inflammatory (3N8Y and 3LN1) receptors supported the in vitro results. Therefore, PCEO or Myristicin might be valuable for developing anti-inflammatory and antimicrobial drugs.

## 1. Introduction

*Petroselinum crispum* (*P. crispum*) is commonly known as parsley. It belongs to the genus Petroselinum and the family Apiaceae (Umbelliferae). It is a native herb in many temperate countries [1]. It is a source of biologically active compounds and phytochemical structures used in traditional medicine systems. 

*P. crispum* herbs have a long history of use as essential constituents that can affect traditional medicine for the treatment of skin diseases, otitis, sniffle, kidney stones, gastrointestinal disorders, haemorrhoid, hypertension, cardiac diseases, hepatotoxicity, hyperlipidaemia, and urinary tract infections, and as a food flavour, antitussive agent, diuretic, and anticoagulants in several countries, such as Iran, Turkey, China, Iraq, Spain, Morocco, Italy, Serbia, and Peru [2,3]. Several researchers have reported the presence of Myristicin, apiole, and various others components in the essential oil of *P. crispum* leaves [4,5]. The myristicin-containing essential oil has been reported to have antimicrobial [6] and anti-inflammatory [7]. The compounds found in parsley or other Apiaceae plants such as apiole, β-myrcene, sabinene, *p*-mentha-1,5,8-triene, β-caryophyllene, β-phellandrene, and β-farnesene were reported to have antimicrobial, antioxidant and anti-inflammatory activities [4,8,9]. Methanolic and aqueous extracts of *P*. *crispum* have demonstrated excellent antioxidant [10], antimicrobial [11], anti-platelet [12], and anticancer [13] activities. Other significant volatile compounds such as β-myrcene [14], sabinene [15], *p*-mentha-1,5,8-triene [16], β-caryophyllene [17], and β-farnesene [18] are also reported strong antimicrobial activity. 

Moreover, in vivo experiments performed using different solvent-based extracts have shown good cardiovascular [3], anti-peptic ulcer [19], antidiabetic and hepatoprotective [20], and neuroprotective activities [21]. The in vitro antioxidant activity of the essential oil of Chinese *P. crispum* seeds was reported to be less than that of the standard agents, butylated hydroxytoluene (BHT) and α-tocopherol [22]. The essential oil of parsley seeds has been reported to contain terpenes and phenylpropanoids as the major components [23,24]. 

Although several parsley cultivars are known, the most common cultivar is *P. crispum* Mill. In Saudi Arabia, it is commonly known as Al-bagdunis. It is widely cultivated in the Al-Kharj region or other parts. Previously, it was used in traditional medicine to treat various ailments; however, it is now being used mainly in food preparation [25]. In most herbal plants, the quantity and quality of the phytoconstituents vary in different geographical locations. 

*P. crispum* is widely distributed in the Al-Kharj region of Saudi Arabia. Although various activities have been reported to excess multiple biological effects in different literature, the essential leaf oil of *P. crispum* of Saudi Arabia has not been studied for its significant constituents. This research article discloses the chemical composition, in vitro antioxidant, antimicrobial, and anti-inflammatory activities of *P. crispum* leaf essential oil (PCEO) grown in Saudi Arabia. Furthermore, in this study, the researchers endeavoured to screen the pharmacological activity, drug-likeness properties, and mechanism of action by using in silico Prediction of Activity Spectra of Substances (PASS) prediction, Swiss Absorption, Distribution, Metabolism, and Excretion (ADME), and molecular docking (MD), respectively.

## 2. Results and Discussion

### 2.1. Composition of Essential Oil

**Figure 1:**
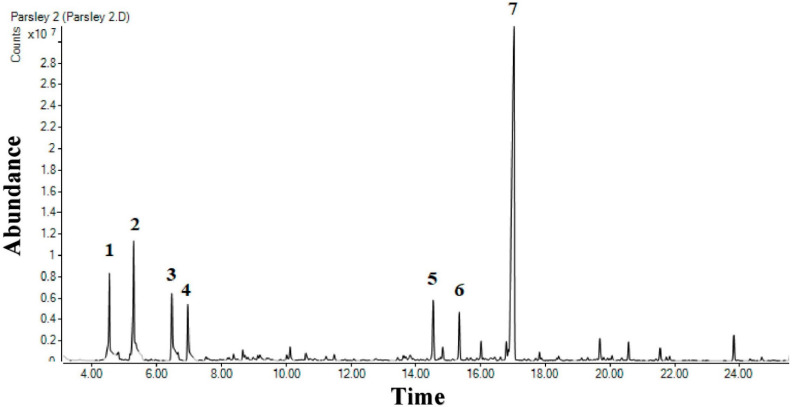
Total ion chromatograms of GC/MS of PCEO using HP-5MS.

The percentage yield of PCEO was approximately 0.08% *w/w*. Table 1 presents the GC–MS analysis of PCEO, and the total ion chromatograms are shown in Figure 1. Volatile compounds in PCEO belong predominantly to the phenylpropanoid class. Myristicin (41.45%) was identified as the significant phenylpropanoids. Monoterpene hydrocarbons represented the second most predominant class, sabinene (9.29%) and β-myrcene (5.98%) were identified as the significant monoterpene hydrocarbons. Sesquiterpene hydrocarbons represented the third primary class, wherein caryophyllene (3.9%) and β-farnesene (2.75%) were identified as the main constituents. The other major compounds were Benzene, (2-methyl-1-propenyl) (5.32%) and *p*-Mentha-1,5,8-triene (4.15%). Total 7 volatile compounds were identified as prominent compounds, collectively representing 72% of the total area 96.02%, and were shown in Table 1. The percentage of other types of volatile compounds such as oxygenated monoterpenes, oxygenated sesquiterpenes, phthalides, and diterpenoids was reported to be less than 2%.

Several leaf essential oil compositions of parsley were identified as *P. crispum* and reported in the literature. Seasonal variations, geographical conditions, and drying conditions play a significant role in causing variations in the chemical composition of leaf essential oil [23]. The effect of seasonal variation was reported in the study. The myristicin content was found to increase drastically, whereas monoterpene content decreased in the leaf oil cultivated in September and harvested in January. Likewise, the apiole content was high in plants grown in January and harvested in April [26]. The effect of geographical variation has been reported in several studies. Farouk and co-workers examined the leaves of parsley from Saudi Arabia (Madinah) and Egypt. They reported variations in the contents of Myristicin, 1,3,8-*p*-menthatriene, β-phellandrene, and myrcene between the leaf oils of Saudi Arabian (Madinah) and Egyptian cultivars [4].

Mangkoltriluk et al. [27] studied the chemical composition of leaf oil from Australian cultivars (Rockdale, New South Wales) and showed the absence of Myristicin. Several studies have confirmed that the variations in the composition depend upon the geographical conditions. For example, cultivars from regions such as South Carolina, California, Greece, and Mexico consist of different concentrations of Myristicin, β-phellandrene, 1,3,8-p-menthatriene, apiole, and *p*-cymene [5,28]. The seasonal variations and drying conditions also play an essential role in causing variations in the chemical composition of PCEO. Lechtenberg et al. analysed two dried samples using different methods. They showed that one of the samples was rich in Myristicin, apiole, and myrcene, whereas another was rich only in Myristicin [29]. In the present study, we identified Myristicin, sabinene, and p-myrcene as the major compounds in the essential leaf oil of *P. crispum* cultivated in the Al-Kharj region. The oil compositions were different from other parts of Saudi Arabia (Madinah), Egypt, Europe, and other countries.

### 2.2. Antimicrobial Activity

The antimicrobial activity of PCEO was evaluated using the agar diffusion assay, and the results are shown in Table 2. The highest antimicrobial activity was observed against fungus Candida albicans (19.4 ± 0.08 mm), at 20 mg/mL followed by gram-positive bacteria Staphylococcus aureus (17.86 ± 0.09 mm), Bacillus subtilis (15.73 ± 0.04 mm) and gram-negative bacteria Klebsiella pneumoniae (9.43 ± 0.09 mm) and Escherichia coli (8.67 ± 0.12). The MIC value for C. albicans, S. aureus, and B. subtilis was 1.25, 2.5, and 2.5 mg/mL, respectively.

The essential oil of *P. crispum* leaves been reported to possess several biological activities. The chemopreventive effects of parsley leaf oil were demonstrated in a study, and the potential cancer-preventive effects were attributed mainly to the presence of Myristicin [30]. Various other *P. crispum* has been reported in studies [31,32]. Myristicin was found to be the principal constituent of PCEO. It was reported to exert several biological effects such as insecticidal, hepatoprotective, anxiolytic, and chemopreventive effects such as the monoamine oxidase inhibitory effect [5]. The antimicrobial activity of *P. crispum* leaf extracts against gram-positive bacteria, gram-negative bacteria, and fungi has been reported [33]. In the present study, we report a superior activity of PCEO against *C. albicans* and *S. aureus*, which may be due to the presence of Myristicin [34]. The company of Myristicin may hasten the antibacterial effects on both gram-positive and gram-negative bacteria [35]. Myristicin also demonstrated sound antifungal effects in a study [36].

As well as myristicin, other compounds such as β-myrcene, sabinene, *p*-mentha-1,5,8-trianes, β-caryophyllene, and β-farnesene demonstrate antibacterial activity as well. Wang et al. [14], studied the antimicrobial activity of myrcene and found the compound to be effective against various microbes. Joshi et al. [37] studied the antimicrobial activity of *Alpinia nutans* containing sabinene as a major aromatic compound and found they are active against *E. coli* and *S. aureus*. Alipour et al. [16], studied the antimicrobial activity of *Ferula cupularis*, which contained p-mentha-1,5,8-triene along with other components, and found that it was effective against both Gram-positive and Gram-negative bacteria. Dahham et al. [17], tested the antimicrobial activity of β-caryophyllene against bacterial and fungal agents and found strong antimicrobial properties. Cherehregani et al. [18], investigated the properties of *Tripleurospermum disci-forme*, which contains β-farnesene as a major volatile compound, which showed good antimicrobial effects against *Staphylococcus* spp. and *Bacillus* spp.

### 2.3. Antioxidant Activity of PCEO

The DPPH-induced free radical scavenging and ferric reducing properties were used to determine the antioxidant activities of PCEO, and the outcomes are shown in Table 3. For PCEO at 5 mg/mL, the increase in the percentage inhibition of DPPH-induced free radical scavenging activity and proliferation in absorbance in the ferric chloride reducing assay was found to be 68.42% ± 0.27% and 0.517 ± 0.01, respectively, which were lower than those of the standard (ascorbic acid).

The antioxidant potential of *P. crispum* leaf extracts has been reported in previous studies [22,38]. In the present study, the antioxidant activity was poor compared to ascorbic acid, which may be due to the weaker antioxidant potential of Myristicin or other volatile chemicals. However, the parsley essential oil containing myristicin and apiol as major compounds demonstrated good antioxidant potential in a study [22]. Myristicin or other chemicals in the oil accounts for its antioxidant potential [36,39]. In addition to myristicin, other important compounds such as β-myrcene, sabinene, and β-caryophyllene also exhibited good antioxidant activity. Ciftci et al. [40], demonstrated the antioxidant activity of myrcene and found that it had good antioxidant properties. Joshi et al. [37], studied the antioxidant activity of *Alpinia nutans*, which contains sabinene as a major component. Dahham et al. [17] studied the antioxidant properties of β-caryophyllene using by using DPPH free radical scavenging and Ferric reducing models and found that it exhibited strong antioxidant effects. Afoulous et al. [41], studied the antioxidant activity of *Cedrelopsis grevei* that contains β-farnesene as a major volatile compound, and reported poor antioxidant activity.

### 2.4. Anti-Inflammatory Activity

**Figure 2:**
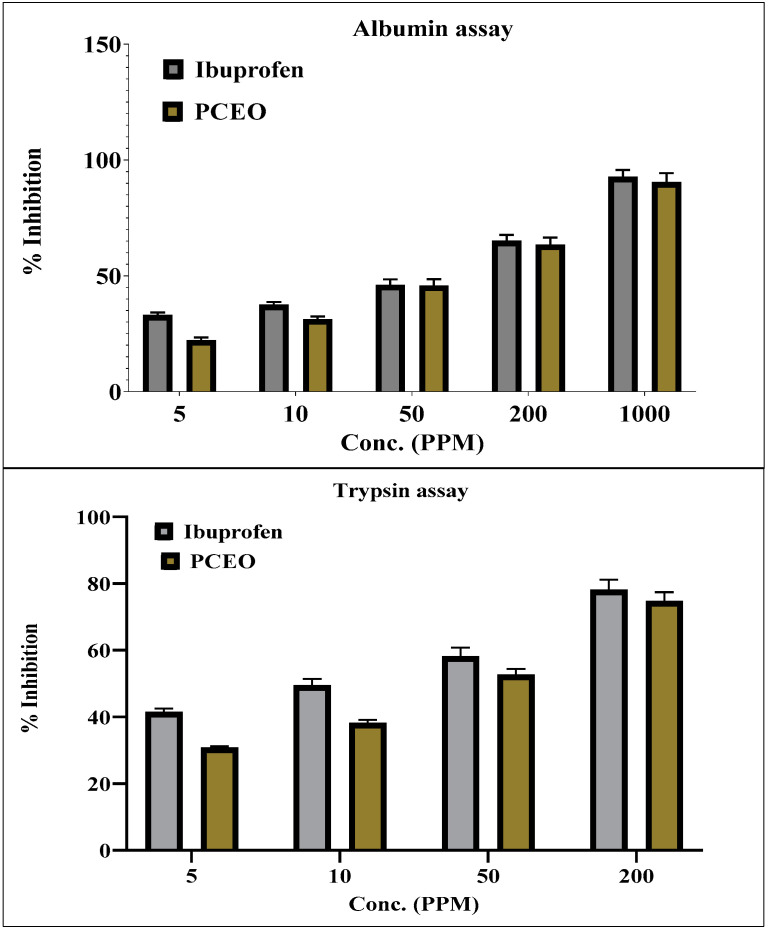
Anti-inflammatory activity of *P.* *crispum* leaves essential oil (PCEO).

The anti-inflammatory activity of PCEO assessed through egg albumin- and trypsin-induced inflammation was found to be excellent compared with that of Ibuprofen, as shown in Figure 2. The percentage inhibition of egg albumin-induced inflammation ranged from 22.2% to 90.4% at the concentration range of 5–1000 PPM, whereas standard Ibuprofen ranged from 32.8% to 91.7% at the same concentration. The percentage inhibition of trypsin-induced inflammation increased from 8.4% to 74.7% at the concentration range of 5–200 PPM, whereas that of Ibuprofen ranged from 74.7% to 76.5% at the same concentration. PCEO displayed nearly similar anti-inflammatory potential in both assays and standard (Ibuprofen).

Studies have also reported the anti-inflammatory potential of *P. crispum* leaves [19]. In the present study, we report the excellent anti-inflammatory activity of PCEO against both egg albumin- and trypsin-induced inflammation, and these effects may be due to the presence of Myristicin. Myristicin accounts for the anti-inflammatory effects. Studies have shown that the anti-inflammatory effects are due to the inhibition of calcium, nitric oxide, cytokines, chemokine, and growth factor synthesis [7,42].

### 2.5. PASS and ADME Prediction

**Figure 3:**
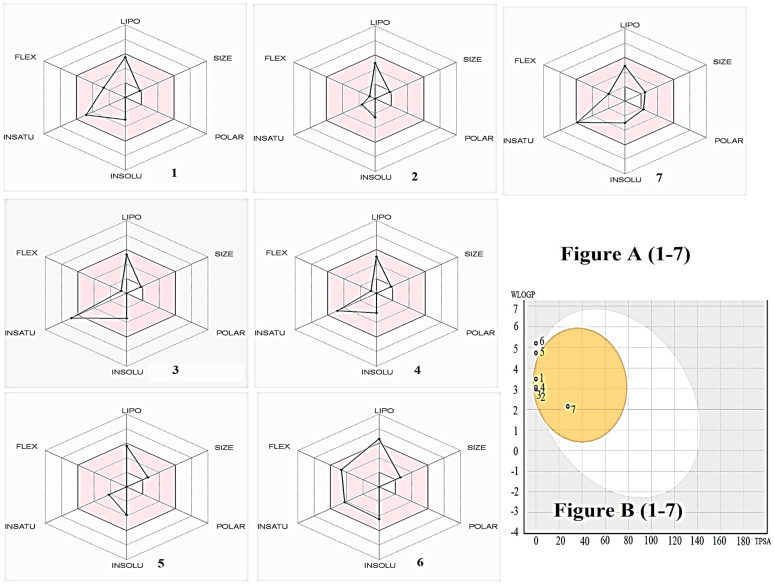
(**A**): bioavailability radar, (**B**): Predicted BOILED-Egg diagram of β-Myrcene (**1**), Sabinene (**2**), Benzene, (2-methyl-1-propenyl) (**3**), *p*-Mentha-1,5,8-triene (**4**), β-Caryophyllene (**5**), β-Farnesene (**6**), Myristicin (**7**) and using Swiss ADME software.

The primary compound of PCEO, Myristicin (1,3-Benzodioxole, 4-methoxy-6-(2-propenyl) ≈ 41%), along with six other compounds, was selected for PASS and ADME prediction studies. The SMILES format of Myristicin was chosen using the ChemBioDraw web tool [43] and then simulated using the PASS prediction web tool [44] and ADME web tool [45]. Table 4 presents the results of PASS and ADME prediction studies. The analysis of the bioavailability radar graph and BOILED-Egg assay of Myristicin and other compounds that predicted drug likeness and bioavailability properties is represented in Figure 3.

The PASS prediction of the major PCEO compound, Myristicin, was performed to predict antioxidant, antimicrobial, and anti-inflammatory activities. Myristicin displayed the “Pa” values of 0.328, 0.265, 0.36, and 0.217 for spasmolytic, anti-inflammatory, antifungal, antioxidant, and antibacterial potential, respectively.

The PASS predictions of the other compounds, β-myrcene, sabinene, benzene, (2-methyl-1-propenyl), *p*-mentha-1,5,8-triene, β-caryophyllene, and β-farnesene, were performed to predict antioxidant, antimicrobial, antifungal, and anti-inflammatory activities. All compounds displayed the significant “Pa” values ranging from anti-inflammatory (0.297–0.853), antifungal (0.256–0.584), antioxidant (0.144–0.497), and antibacterial (0.201–0.437) potential (Table 4). We predicted that all significant compounds exhibit excellent anti-inflammatory, good antifungal, and low antibacterial and antioxidant properties from these results.

ADME web tool is valuable for understanding the physicochemical, pharmacokinetic, and drug-likeness properties of compounds. Drug-likeness properties of selected compounds can be evaluated through the topological polar surface area (TPSA), several rotatable bonds, hydrogen bond acceptor and donors, and molar refractivity. Other parameters, such as Lipinski’s and bioactivity score, were used to predict drug likeness properties. The prediction results showed that all significant compounds with a bioactivity score of 0.55 obeyed all the drug-likeness rules without any violations, and synthetic accessibility (1.47–4.51) showed a straightforward synthetic route. The lipophilicity values of all selected compounds showed that it is soluble in water.

The pharmacokinetic limits such as skin permeation, absorption, distribution, metabolism, and excretion were also predicted. The graph of bioavailability radar, which signifies the pharmacokinetics, physicochemical, and drug-likeness properties of volatile compounds present in PCEO, was analysed using Swiss-ADME software [45]; the outcomes are reported in Figure 3A(1–7). The BOILED-Egg method was used to predict the ability of selected compounds for GI absorption and passive diffusion through the blood-brain barrier (BBB). The BOILED-Egg prediction results “Figure 3B(1–7)” in a study showed that except β-caryophyllene and β-farnesene, all other compounds could diffuse through the BBB and possess gastrointestinal absorption properties [46]. Skin permeability parameters (log Kp) indicated that all selected compounds have good permeability value [46]. Glycoprotein (P-gp) has a significant role in drug absorption and dispersion, and the selected compounds were not found to be a substrate of P-gp. Cytochrome P450 (CYP) enzymes and molecular interactions are essential for drug elimination; Myristicin inhibits CYP1A2, β-farnesene inhibits CYP2C9, β-caryophyllene inhibits CYP2C19 and CYP2C9, and p-mentha-1,5,8-triene inhibits CYP2C9 iso-enzymes, resulting in adverse effects and drug-drug interaction [47].

### 2.6. Molecular Docking Studies

**Figure 4:**
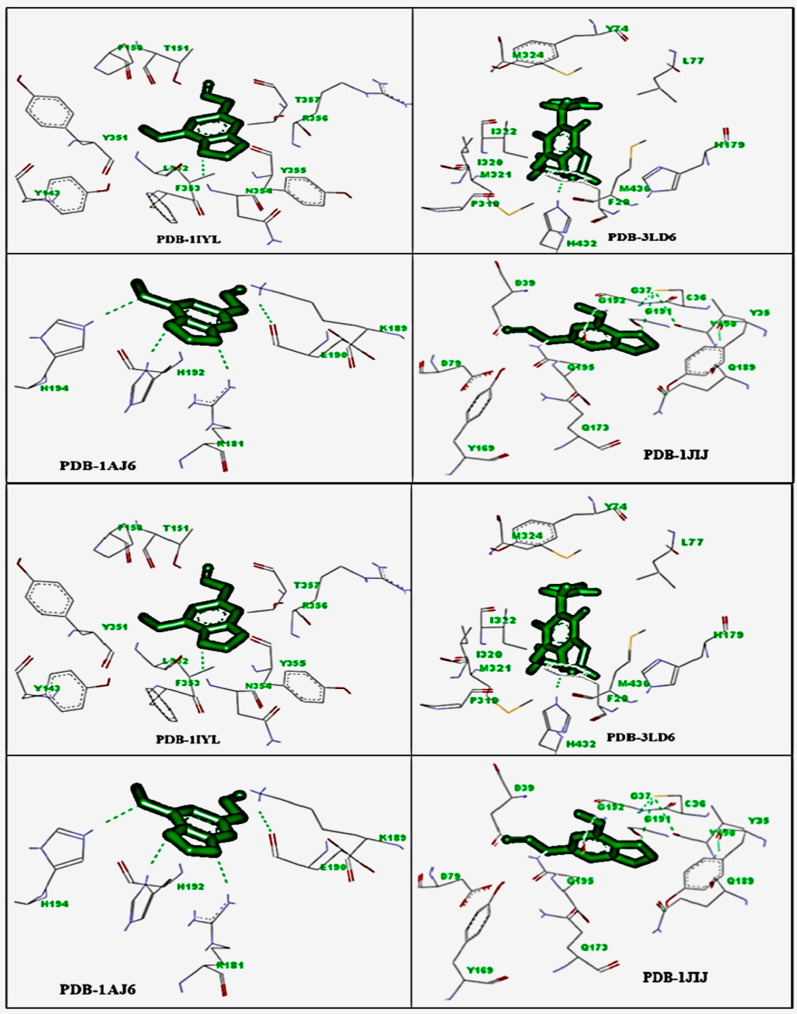
Interaction of 1IYL, 3LD6, 1AJ6, 1JIJ, 3N8Y, 3LN1, 3NM8, and 1HD2 protein with Ligand myristicin.

Among all analysed volatile compounds, Myristicin was found as major metabolites; hence, the molecular docking studies were carried out for Myristicin only in the present study. In silico docking simulations of Myristicin were performed on targets such as antifungal (PDB: 1IYL, 3LD6), antibacterial (PDB: 1AJ6 and 1JIJ), anti-inflammatory (3N8Y and 3LN1), and antioxidant (3NM8 and 1HD2) receptors. Table 5 shows the binding energy (ΔG: kcal/mol) and inhibition constant (Ki: µM). Figure 4 shows the interaction of Myristicin with antifungal and antibacterial, anti-inflammatory, and antioxidant receptors.

Myristicin has been reported as the primary compound responsible for antifungal activity in studies [36,48]. In the present study, two antifungal targets, N-myristoyl transferase (1IYK) and cytochrome P450 14α-demethylase (3LD6), were docked with Myristicin. The interaction of Myristicin with N-myristoyl transferase displayed high binding energy (ΔG −8.50 kcal/mol) and low inhibition constant (327.76 µM), with ASN354 residues conferring H-bonding stability to the complex. Similarly, when Myristicin was docked with cytochrome P450 14α-demethylase (3LD6), the binding energy was −8.38 kcal/mol, the inhibition constant was 423.76 µM, and HIS432 residue was found to confer H-bonding stability to the complex. The enzyme N-myristoyl transferase is involved in the translocation of fatty acids for the structural modification of membranes [49]. In contrast, cytochrome P450 14α-demethylase contributes to ergosterol biosynthesis in the fungal membrane [50]. Based on these results, we predicted that the inhibition of fatty acid translocation and ergosterol biosynthesis accounts for the antifungal activity of Myristicin.

When Myristicin was simulated with the antibacterial target DNA gyrase B kinase, the binding energy was −7.37 kcal/mol, the inhibition constant was 523.76 µM, and H-bonding stability of the complex was provided by ARG181, HIS192, and HIS194 residues. Similarly, when Myristicin was docked with tyrosyl-tRNA synthetase, the binding energy was −7.55 kcal/mol. The inhibition constant was 480.43 µM, and the H-bonding stability of the complex was conferred by CYS36 residue. From these findings, we predicted that Myristicin possesses antibacterial potential. The enzyme DNA gyrase B kinase plays a crucial role in inhibiting bacterial chromosome segregation and transcription and replication of DNA; therefore, this target is appealing for developing antibacterial drugs [51].

Similarly, the enzyme tyrosyl-tRNA synthetase, a member of the aminoacyl-tRNA synthetase family, is involved in the translation process during protein synthesis, and inhibitors of this enzyme could be an interesting target for the development of novel antimicrobial agents [52]. Based on these results, we predicted that the inhibition of the enzymes DNA gyrase B kinase and tyrosyl-tRNA synthetase accounts for the antibacterial effects of Myristicin. Exploring the anti-inflammatory mechanism of Myristicin was first simulated with COX-1. The results obtained are as follows: binding energy, −8.15 kcal/mol; inhibition constant, 543.67 µM; and the residue conferring H-bonding stability to the complex, THR181.

Myristicin was then docked with COX-2, and the results are as follows: binding energy, −8.79 kcal/mol; inhibition constant, 334.98 µM; and the residue conferring H-bonding stability to the complex, ALA125. Based on these findings, we predicted that Myristicin has anti-inflammatory potential. The enzyme COX-1 plays a crucial role in the synthesis of PGE_2_, PGI_2,_ TxA_2,_ and PGF_2α_, whereas COX-2 is a predominantly an inducible enzyme, which is considered mainly responsible for the production of prostanoids in inflammation; overall, COX-2 plays a significant role in inflammation, and COX-1 contributes in the initial stage of inflammation. The enzyme COX-1 is involved more in homeostasis than in inflammation, whereas COX-2 is involved more in inflammation. [53]. Based on these results, we predicted that Myristicin first inhibits COX-2 and then inhibits COX-1, and the inhibition of both COX-1 and COX-2 enzymes accounts for its anti-inflammatory activity.

To elucidate the antioxidant mechanism, Myristicin was simulated with tyrosinase enzyme. The predicted results were: binding energy, −6.93 kcal/mol; inhibition constant, 765.64 µM, and a residue conferring H-bonding stability to the complex, ARG162. The tyrosinase enzyme inhibition has played a key role to avert biosynthesis of melanin in skin and hydroxylation of l-tyrosine to 3,4-dihydroxyphenylalanine (DOPA) and oxidation of DOPA to dopaquinone [54]. Therefore, tyrosinase inhibitors are an attractive target for the evaluation of pigmentation disorders and antioxidant potential. Similarly, when Myristicin was docked with the human peroxiredoxin 5 receptor, the following results were obtained: binding energy, −7.64 kcal/mol; inhibition constant, 549.74 µM; and the residue conferring H-bonding stability to the complex, GLY92. We predicted that Myristicin has a low antioxidant potential based on these findings. The antioxidant potential is mainly due to the inhibition of the human peroxiredoxin 5 receptor [55].

## 3. Materials and Methods

### 3.1. Extraction of Essential Oil

Fresh leaves of *P. crispum* were collected in February 2018 from the local market of Al-Kharj (Central Region, Kingdom of Saudi Arabia, KSA). Samples were authenticated and deposited at the herbarium of the Pharmacognosy Department, College of Pharmacy, Prince Sattam Bin Abdulaziz University, Al-Kharj, KSA. The voucher specimen number was PSAU-CPH-O2-2018. PCEO was isolated and purified using the previously described method [56].

### 3.2. Gas Chromatography-Mass Spectrometry

Gas chromatography-mass spectrometry (GC–MS) analysis of PCEO was performed using Agilent-7890 mass spectrometer coupled with HP5-MS (Agilent Technologies, Santa Clara, CA, USA) and capillary column (30 m × 0.25 mm; i.d., 0.25 μm). One microliter of the essential oil (10% in acetone) was injected into the GC–MS instrument in a splitless mode. The injector temperature and flow rate were maintained at 280 °C and 1 mL/min, respectively, and helium (99.999%) was used as the carrier gas. Initially, the column temperature and flow rate were set at 40 °C and 5 °C/min, respectively, for 2 min. Then, the temperature was increased to 70 °C while maintaining the same flow rate for 5 min. Finally, the temperature was increased to 290 °C at the rate of 3 °C/min, and this temperature was maintained for 5 min. The operating parameters of MS are as follows: mode, electron impact (EI); ionisation voltage, 70 eV; quadrupole temperature, 150 °C; ion source temperature, 180 °C; scan/mass range, 30–600 amu; and scan rate, 0.32 s/scan. The PCEO was extracted three times. Retention indices (RIs) were calculated by using retention times on homologous series of *n*-alkanes (C8-C40) as well as by following Vandendool and Kratz’s linear equation [57]. The retention indices and mass spectra of individual components were compared with those found in GC-MS libraries (National Institute of Standards and Technology, 2017) and Adams’s [58].

### 3.3. Antimicrobial Activity

The antimicrobial activity against a total of five microbial species, namely, *S. aureus* (ATCC 25923), *B. subtilis* (ATCC 11774), *E. coli* (ATCC 11229), *K. pneumoniae* (NCTC 9633), and *C. albicans* (ATCC 1023), was determined. Mueller Hinton Agar/broth (MHA, Hi-Media) and potato dextrose agar/broth (PDA, Hi-Media) were used in the present study. All selected strains were obtained from the Department of Pharmaceutics, College of Pharmacy, PSAU, Al-Kharj, Saudi Arabia. The pure strains of bacterial cultures were sub-cultured on MHA, whereas *C. albicans* was cultured on PDA. The antimicrobial properties of PCEO were evaluated using the disc diffusion method, as described by Demo et al. [59], with some modifications. Briefly, 200 μL inoculum of each microorganism was spread separately over solidified MHA Petri dishes, and three Petri plates were prepared for each microorganism. Paper filter disc of 6 mm was impregnated with 50 μL of 5 mg/mL, 10 mg/mL, and 20 mg/mL (DMSO) oil solution. Then, the discs were placed on MHA or PDA plates. A disc saturated with 50μL DMSO for the negative control was used. After placing the disc onto the seeded plates, the plates were incubated in an incubator at 37 °C for 24 h. To observe the growth inhibition zone, the inhibition zone around each disc was measured (in mm) and is reported as mean ± standard deviation (SD) (mm).

The minimum inhibitory concentration (MIC) was determined by placing 20 µL of each dilution (0.025–2 mg/mL in DMSO) on a filter paper disc. The discs were placed on the surface of MHA plates (bacteria) and PDA plates (*C. albicans*) previously inoculated with a 200 µL inoculum. After placing the oil-containing discs onto the plate, the plates were kept at room temperature to allow the diffusion of oil, and then they were incubated. MIC was measured to determine the lowest concentration inhibited the growth.

### 3.4. Antioxidant Activity

In the ferric reducing assay, solutions of trichloroacetic acid (TCA, 10% *w*/*v*), potassium ferricyanide (1%), 0.2 M phosphate buffer (pH 6.6), ferric chloride (0.1%), and standard (Ascorbic Acid) and sample dilutions (1000–10 μg/mL) were prepared. The reaction mixture containing 200 µL sample or standard, 2.5 mL buffer, and 2.5 mL potassium ferricyanide was incubated at 50 °C for 20 min. And the reaction was stopped by adding 2.5 mL TCA. Then, the mixtures were centrifuged at 1000 rpm/10 min; 2.5 mL of the upper layer was separated and mixed with distilled water (2.5 mL) and FeCl_3_ (0.5 mL). Subsequently, the absorbance was measured at 700 nm using a UV–Vis spectrophotometer (Thermo Scientific, Madison, WI, USA).

DPPH (2,2-diphenyl-1-picrylhydrazyl, Sigma-Aldrich) free radical scavenging and ferric chloride reducing ability of PCEO were measured according to Foudah et al. [60]. In the DPPH assay, 10 mL of DPPH methanol solution (1 mmol/L) and dilutions of the standard (Ascorbic Acid) and sample PCEO (5–0.25 mg/mL) were prepared. Afterwards, 100 μL of the standard sample and 1900 μL of DPPH were incubated at 22 °C (in the dark) for 30 min. The inhibition of free radicals was measured after reading the absorbance at 517 nm using a UV spectrophotometer. After measuring the control absorbance (CA) and sample absorption (SA) in the three plates, the percentage (%) of inhibition strength of PCEO was calculated using the following formula:

Scavenging (%) = [CA − SA/CA] × 100.(1)

In the ferric reducing assay, the solutions of trichloroacetic acid (TCA, 10% *w*/*v*), potassium ferricyanide (1%), 0.2 M phosphate buffer (pH 6.6), ferric chloride (0.1%), and standard (Ascorbic Acid) and sample dilutions (5–0.25 mg/mL) were prepared. The reaction mixture containing 200 µL sample or standard, 2.5 mL buffer, and 2.5 mL potassium ferricyanide was incubated at 50 °C for 20 min. And the reaction was stopped by adding 2.5 mL TCA. Then, the mixtures were centrifuged at 1000 rpm/10 min; 2.5 mL of the upper layer was separated and mixed with distilled water (2.5 mL) and FeCl_3_ (0.5 mL). Subsequently, the absorbance was measured at 700 nm using a UV–Vis spectrophotometer.

### 3.5. Anti-Inflammatory Activity

The anti-inflammatory effects of PCEO on egg albumin- and trypsin-induced inflammation were assessed using the methods described by Alam and Singh. Ref.[61] and Gunathilake et al. [62], respectively, with a few modifications.

Briefly, in the egg albumin assay, different dilutions of the sample (5–200 µg/mL) were prepared in phosphate-buffered saline (PBS, pH = 6.8). The reaction mixture containing 100 µL of each dilution, 1000 µL albumin solution (1%), and 1400 µL PBS was incubated for 15 min at 37 °C and then heated in an oven (for 5 min at 72 °C). Subsequently, the absorbance was determined using a spectrophotometer at 660 nm.

For the trypsin assay, the reaction mixture containing 1000 µL of each diluted sample, 0.06 mg trypsin, and 1 mL Tris-HCl was incubated for 15 min at 37 °C. The reaction was stopped by adding 1 mL of casein (0.8%), and the mixture was further incubated for 20 min at 37 °C. Then, 2 mL HClO_4_ (perchloric acid; 70%) was added, and the mixture was centrifuged at 3000 rpm for 5 min. The absorbance (Abs) of the supernatant was measured at 210 nm. Ibuprofen was used as the standard, and a mixture containing all reagents (except sample/standard), following all steps mentioned above, was used as the control. Each experiment was repeated thrice, and the inhibition (%) was determined using the following equation:

Inhibition (%) = [(1 − Abs(sample)/Abs(control) × 100](2)

The results are expressed as mean ± SD of triplicate.

### 3.6. In Silico PASS and ADME Prediction

PASS was used to predict the pharmacological activities of significant compounds detected in PCEO. The selected compounds were first converted into the SMILES format using ChemDraw and then predicted using the PASS online web tool [33]. PASS indicates the probable activity (Pa) and probable inactivity (Pi) of ‘drug-like’ substances [63].

In the pharmacokinetic prediction using ADME, the selected compounds (ligands) were assessed using ADME software, which can predict the physicochemical properties, lipophilicity drug likeness, and toxicity of compounds [45].

### 3.7. Molecular Docking Prediction

Two significant components present in PCEO were selected for the in silico study. The two-dimensional SMILES string of sabinene (CID:18818) and Myristicin (CID: 4276) was accessed from the PubChem database and then converted into the PDB format by using the Babel online tool [64]. Ligands were energetically minimised and optimised using the appropriate force field [65,66].

Docking simulations were performed using AutoDockTools-1.5.6 (ADT) [67]. The selected significant PCEO compounds were subjected to docking with target proteins, namely *N*-myristoyl transferase, PDB: 1IYL [68], cytochrome P450 14α-demethylase, PDB: 3LD6 [69], DNA gyrase B kinase, PDB: 1AJ6 [70], tyrosyl-tRNA synthetase, PDB: 1JIJ [71], COX-1, PDB: 3N8Y [72], COX-2, PDB: 3LN1 [73], tyrosinase enzyme PDB: 3NM8 [74], and human peroxiredoxin 5 receptor, PDB: 1HD2 [75], which were retrieved from the PDB. Docking simulation of proteins and ligands was performed using AutoDockTools-1.5.6 (ADT) to predict their preferential binding affinity in terms of binding energy (ΔG) and inhibition constant (ki). To accomplish ADT execution, various input files such as ligand and proteins, pdbqt ligand gpf, and proteins Dpf were prepared. Adequate spacing between the grids was ensured so that the ligands could move freely inside. The grid size in the x, y, and z axes was drawn at 60 Å and 0.678 Å spacing between the two consecutive grids. A Lamarckian genetic algorithm was used to determine rigid-protein-molecular interactions. Ten conformational runs, 250,000 energy evaluations, and 27,000 generations were adopted during the ADT execution. One of the best conformational poses of ligands showing the least ΔG and ki values was used for further docking analysis [51,76].

### 3.8. Statistical Analysis

All data were statistically evaluated using GraphPad InStat software. The results are articulated either as average or as mean ± SD of experiments conducted in triplicate. Docking complexes were visualised using a DS visualiser.

## 4. Conclusions

This study concludes that Myristicin was the principal phytoconstituent present in PCEO. The in vitro study showed that PCEO has high anti-inflammatory potential. The online theoretical PASS prediction-based studies on Myristicin support the in vitro study results indicating excellent anti-inflammatory, antifungal, and antibacterial potential. Studies using ADME tools showed that Myristicin has drug-like properties. It follows the Lipinski rule, possesses a high GI absorption value, and exerts less on cytochrome (CYP) P450 enzymes. The docking results of myristicin receptor in terms of binding energy and inhibition constant concluded that enzyme inhibition is highly predicted in anti-inflammatory receptors (3LN1 and 3N8Y) and antifungal receptors (1IYL, and 3LD6), followed by antibacterial (1AJ6 and 1JIJ) and antioxidant (1HD2 and 3NM8) receptors. Overall, the present in vitro study results were supported by the in silico docking study results related to Myristicin’s primary compound. Therefore, Myristicin may be a valuable candidate for the development of COX-2 inhibitors, antifungal agents (N-myristoyl transferase and cytochrome P450 14α-demethylase inhibitors), antimicrobial agents (tyrosyl-tRNA synthetase and DNA gyrase B kinase inhibitors), and antioxidant agents (human peroxiredoxin 5 receptor inhibitors).

## Figures and Tables

**Table 1:** Chemical composition of PCEO analysed using GC-MS.

Numbers	Metabolites	LRI (Exp.)	LRI (Lit.)	Area %
**1.**	**β-** **Myrcene (1)**	971	969	**5.98**
2.	α-Phellandrene	1001	1004	0.44
3.	*cis*-3-Hexenyl Acetate	1008	1009	0.36
4.	1,5,5-Trimethyl-6-methylene-cyclohexene	1338	1338	0.14
5.	β-Cymene	1020	1021	0.30
**6.**	**Sabinene (2)**	975	977	**9.29**
7.	γ-Terpinene	1059	1062	0.12
**8.**	**Benzene, (2-methyl-1-propenyl) (3)**	1072	1067	**5.32**
9.	Linalool	1100	1104	0.60
**10.**	***p*** **-Mentha-1,5,8-triene (4)**	1112	1112	**4.15**
11.	4-Isopropyl-1-methyl-2-cyclohexen-1-ol	1140	1144	0.20
12.	*trans*-Borneol	1167	1165	0.13
13.	Menthol	1168	1169	0.34
14.	2-Acetyltoluene	1172	1171	0.55
15.	Kryptone	1186	1187	0.32
16.	α-Terpineol	1185	1187	0.16
17.	L-Perillaldehyde	1281	1285	0.21
18.	Methyl-*p*-tolylcarbinol	1090	1098	0.27
19.	1-Methyl-4-(1-methylpropyl)-benzene	1097	1100	0.48
20.	4,7-Dimethylbenzofuran	1224	1220	0.12
21.	Pulegone	1243	1244	0.27
22.	2,3,5,6-Tetramethylphenol	1312	1319	0.78
23.	5-Decen-1-ol, (*Z*)-	1201	-	0.55
24.	1-Decanol	1273	1272	0.19
25.	Carvacrol	1299	1298	0.73
26.	2,6,8-Trimethylbicyclo[4.2.0]oct-2-ene-1,8-diol	1303	-	0.35
27.	Copaene	1372	1376	0.20
28.	β-Damascenone	1385	1384	0.32
29.	β-Bourbonene	1386	1384	0.30
30.	β-Elemene	1392	1394	0.63
31.	4-(2,6,6-Trimethyl-1,3-cyclohexadien-1-yl)-2-butanone	1425	1424	0.16
**32.**	**β-Caryophyllene (5)**	1426	1428	**3.96**
33.	γ-Elemene	1435	1432	0.89
**34.**	**β-Farnesene (6)**	1458	1459	**2.76**
35.	(+)-epi-Bicyclosesquiphellandrene	1499	1498	0.20
36.	γ-Muurolene	1481	1485	0.18
37.	β-Copaen-4α-ol	1579	1570	1.51
38.	β-Ionone	1486	1488	0.15
39.	β-Selinene	1488	1489	0.22
40.	α-Cedrene	1409	1409	0.37
41.	α-Muurolene	1492	1497	0.29
42.	β-Bisabolene	1509	1506	0.3
43.	γ-Cadinene	1513	1511	1.19
**44.**	**Myristicin (7)**	1523	1519	**41.45**
45.	α-Cadinene	1546	1544	0.17
46.	Benzene,1,2,3-trimethoxy-5-(2-propenyl)-	1556	1559	0.16
47.	Germacrene B	1558	1561	0.49
48.	Copaen-15-ol	1572	1574	0.19
49.	Caryophyllene oxide	1583	1578	0.41
50.	α-Amorphene	1490	1494	0.33
51.	Caryophylla-4(12),8(13)-dien-5β-ol	1640	1644	0.11
52.	*T*-Cadinol	1642	1644	1.39
53.	butylphthalide	1656	1658	0.12
54.	α-Cadinol	1658	1660	0.11
55.	Isoaromadendrene epoxide	1577	1579	0.25
56.	Ledene oxide-(II)	1680	1682	0.19
57.	Apiol	1691	1696	1.03
58.	Senkyunolide	1729	1729	0.12
59.	*trans*-Sedanolide	1737	1735	0.69
60.	*(E)*-Ligustilide	1807	1809	0.21
61.	4,8-Epithioazulene	1743	1744	0.26
62.	Phytol	2117	2119	1.58
	Total % Area			95.24%

Values of area % represented the average of three independently extracted PCEO. β-Myrcene (**1**), Sabinene (**2**), Benzene, (2-methyl-1-propenyl) (**3**), *p*-Mentha-1,5,8-triene (**4**), β-Caryophyllene (**5**), β-Farnesene (**6**), and Myristicin (**7**) were identified as significant metabolites.

**Table 2:** Zone inhibition (mm) and Minimum Inhibitory Concentration (MIC) analysis of *P. crispum* leaves essential oil (PCEO).

Microorganisms	Zone of Inhibition (mm)			MIC (mg/mL)
	5 mg/mL	10 mg/mL	20 mg/mL	
*S. aureus*	9.7 ± 0.14	13.7 ± 0.08	17.86 ± 0.09	2.5
*B. subtilis*	8.63 ± 0.12	12.73 ± 0.05	15.73 ± 0.04	2.5
*E. coli*	NI	7.33 ± 0.09	8.67 ± 0.12	<5
*K. pneumonia*	NI	9.3 ± 0.08	9.43 ± 0.09	<5
*C. albicans*	11.63 ± 0.12	15.73 ± 0.04	19.4 ± 0.08	1.25

Values showed the means of three (*n* = 3) independent replicates ± SD.

**Table 3:** Antioxidant activity of *P.* *crispum* leaves essential oil (PCEO), using DPPH and FeCl_3_ methods.

Conc. (mg/mL)	DPPH Assay, % Inhibition		Ferric Chloride Assay, Absorbance	
	Ascorbic Acid	PCEO	Ascorbic Acid	PCEO
**0.25**	95.09 ± 0.56	3.72 ± 0.05	1.104 ± 0.02	0.086 ± 0.02
**0.5**	95.48 ± 0.11	8.4 ± 0.07	1.557 ± 0.03	0.117 ± 0.01
**1**	96.93 ± 0.23	28.62 ± 0.13	1.673 ± 0.08	0.180 ± 0.003
**2.5**	98.09 ± 0.36	49.85 ± 0.18	1.837 ± 0.05	0.300 ± 0.01
**5**	98.53 ± 0.44	68.42 ± 0.27	1.901 ± 0.03	0.517 ± 0.01

Values showed the means of three (*n* = 3) independent replicates ± SD.

**Table 4:** In silico PASS and ADME Prediction of PCEO major metabolites.

Prediction	1	2	3	4	5	6	7
**PASS Prediction (Pa/Pi)**							
Anti-inflammatory	0.297 /0.073	0.853 /0.005	0.357 /0.119	0.282 /0.178	0.437 /0.023	0.326 /0.139	0.382 /0.105
Antioxidant	0.470 /0.008	-	0.353 /0.016	0.144 /0.110	0.174 /0.075	0.497 /0.007	0.360 /0.016
Anti-fungal	0.584/0.020	0.340 /0.066	0.399 /0.050	0.517 /0.013	0.582 /0.020	0.607 /0.018	0.256 /0.103
Antibacterial	0.398 /0.030	0.201 /0.117	0.293 /0.063	0.431 /0.024	0.437 /0.023	0.415 /0.027	0.217 /0.103
**ADME Prediction**							
Physiochemical Properties							
TPSA (Å):	0.00 Å²	0.00 Å²	0.00 Å²	0.00 Å²	0.00 Å²	0.00 Å²	27.69 Å²
Molar refractivity	48.76	45.22	46.15	46.65	68.78	72.32	53.10
**Drug Likeness Prediction**							
Bioactivity Score	0.55	0.55	0.55	0.55	0.55	0.55	0.55
Synthetic accessibility	2.85	2.87	1.47	4.10	4.51	3.42	2.40
**Absorption Parameters Prediction**							
Consensus Log S	−3.88	−2.76	−3.50	−2.99	−4.10	−5.81	−3.18
Consensus Log Po/w:	3.43	3.25	3.33	2.94	4.24	4.97	2.49
Solubility Class	Soluble	Soluble	Soluble	Soluble	Soluble	Soluble	Soluble
**Distribution Parameters Prediction**							
Log Kp (cm/s)	−4.17	−4.94	−4.40	−4.77	−4.44	−3.27	−5.39
GI absorption	Low	Low	Low	Low	Low	Low	High
BBB permeant	Y	Y	Y	Y	No	No	Y
**Metabolism Parameters Prediction**							
P-glycoprotein substrate	No	No	No	No	No	No	No
CYP1A2, CYP2C19, CYP2C9 CYP2D6 and CYP3A4 inhibitors	No	No	No	No (accept)	No (Accept CYP2C19 and CYP2C9 inhibitors)	No (Accept CYP2C9 inhibitor)	No (accept CYP1A2 inhibitor)

Where “Pa” is probable activity, “Pi” is probable inactivity, “Å²” polar surface area, and β-Myrcene (**1**), Sabinene (**2**), Benzene, (2-methyl-1-propenyl) (**3**), *p*-Mentha-1,5,8-triene (**4**), β-Caryophyllene (**5**), β-Farnesene (**6**), Myristicin (**7**).

**Table 5:** The binding energy (ΔG: kcal/mol) and inhibition constant (Ki: µM) for Myristicin with target proteins.

Targets Proteins (PDB)	ΔG	Ki	H-Bonds	Residues H-Bonding
*N*-myristoyl transferase (1IYL)	−8.50	327.76	1	ASN354
Cytochrome P450 14α-demethylase (3LD6)	−8.38	423.76	1	HIS 432
DNA gyrase B kinase (1AJ6)	−7.37	523.76	3	ARG181, HIS192, HIS194
tyrosyl-tRNA synthetase (1JIJ)	−7.55	480.43	1	CYS36
COX-1 (3N8Y)	−8.15	543.67	1	THR181
COX-2 (3LN1)	−8.79	334.98	1	ALA125
Tyrosinase enzyme (3NM8)	−6.93	765.64	1	ARG162
Human Peroxiredoxin 5 receptor (1HD2)	−7.64	549.74	1	GLY92

## Data Availability

The data presented in this study are available in this article.

## Sample Availability

Samples of the plants are available from the authors.
